# Molecular mechanism of bisphenol A in promoting esophageal carcinoma based on network toxicology and molecular docking

**DOI:** 10.1097/MD.0000000000046829

**Published:** 2025-12-26

**Authors:** Ming Hou, Hanbing Jia, Chongyang Liu, Cheng Wang

**Affiliations:** a Department of Thoracic Surgery, The Second Hospital & Clinical Medical School, Lanzhou University, Lanzhou, China; b Department of Clinical Medicine, The First Clinical Medical College, Lanzhou University, Lanzhou, Gansu, China.

**Keywords:** bisphenol A, endocrine disruptor, esophageal carcinoma, HSP90AA1, HSP90AB1, molecular docking, mutation analysis, network toxicology, TCGA

## Abstract

Bisphenol A (BPA) is a pervasive endocrine-disrupting chemical with estrogenic activity and has been implicated in the development of multiple malignancies. However, its molecular mechanisms in esophageal carcinoma (ESCA) remain unclear. This study aimed to elucidate the potential oncogenic pathways through which BPA contributes to ESCA progression. Network toxicology was applied to collect BPA-related targets and ESCA-associated genes from multiple public databases. Overlapping targets were identified for further protein–protein interaction (PPI) and enrichment analyses to investigate functional pathways. Molecular docking was performed to assess binding affinities between BPA and core targets. The Cancer Genome Atlas (TCGA) was used for expression and survival validation, while mutation profiles were examined via cBioPortal. A total of 100 BPA-related targets and nearly 50,000 ESCA-associated genes were retrieved, yielding 95 overlapping targets. PPI network analysis and enrichment results highlighted HSP90AA1 and HSP90AB1 as central hub genes associated with protein kinase regulation, telomerase activity, and immune–inflammatory signaling pathways. Molecular docking confirmed strong binding affinities between BPA and HSP90AA1/HSP90AB1 (−7.5 and −7.0 kcal/mol, respectively). TCGA analyses showed that both genes were significantly upregulated in ESCA tissues, and high expression correlated with poorer overall survival. Mutation profiling indicated that HSP90AB1 exhibited a higher alteration frequency (13%), predominantly driven by gene amplification. This integrative multi-omics analysis provides compelling evidence that BPA may facilitate ESCA progression through HSP90AA1/HSP90AB1-mediated oncogenic and immune–inflammatory pathways. These findings deepen understanding of environmental carcinogenesis and suggest potential molecular targets for ESCA prevention and treatment.

## 1. Introduction

Bisphenol A (BPA) is a widely distributed environmental endocrine disruptor and a commonly used synthetic organic compound. Humans are mainly exposed to BPA through dietary intake (migration from food packaging and containers), as well as through dermal contact and inhalation.^[1]^ BPA is extensively used in the manufacture of polycarbonate plastics and epoxy resins, and is commonly found in water bottles, food containers, metal can coatings, and other daily-use products.^[2]^ Due to its estrogen-like activity, BPA can be detected in blood, urine, placenta, and breast milk.^[3]^ Both epidemiological and experimental studies have suggested that BPA exposure is associated with reproductive, immune, neurological, and endocrine disorders, and exhibits hepatotoxic, immunotoxic, mutagenic, and potentially carcinogenic effects.^[4–6]^ Recent studies have demonstrated that BPA influences tumor cell growth, migration, apoptosis, and drug resistance by modulating multiple signaling pathways,^[7]^ and can activate mutant androgen receptors to promote prostate cancer cell proliferation.^[8]^

Esophageal carcinoma (ESCA) is a common malignant tumor, ranking 11th in incidence and 7th in mortality worldwide.^[9]^ Its occurrence and progression are not only associated with environmental factors such as alcohol consumption, smoking, nutritional deficiency, and gastroesophageal reflux disease, but are also closely linked to genetic heterogeneity, epigenetic abnormalities, and dysregulation of multiple signaling pathways.^[10]^ Previous studies have shown that HSP90AA1 is significantly overexpressed in ESCA, functioning as a super-enhancer–regulated gene that drives tumor proliferation and migration, and its high expression is closely associated with poor prognosis.^[11]^ HSP90AB1, on the other hand, promotes cell proliferation, migration, and drug resistance by stabilizing and activating key signaling pathways such as MAPK/ERK and PI3K/AKT, and its high expression is similarly associated with poor prognosis.^[12]^ Moreover, HSP90AB1 has been incorporated into immune-related^[13]^ and autophagy-related gene models^[14]^, suggesting that it also plays a critical role in immune regulation and autophagy, thereby enhancing tumor cell survival and accelerating disease progression.

**Figure 1.**
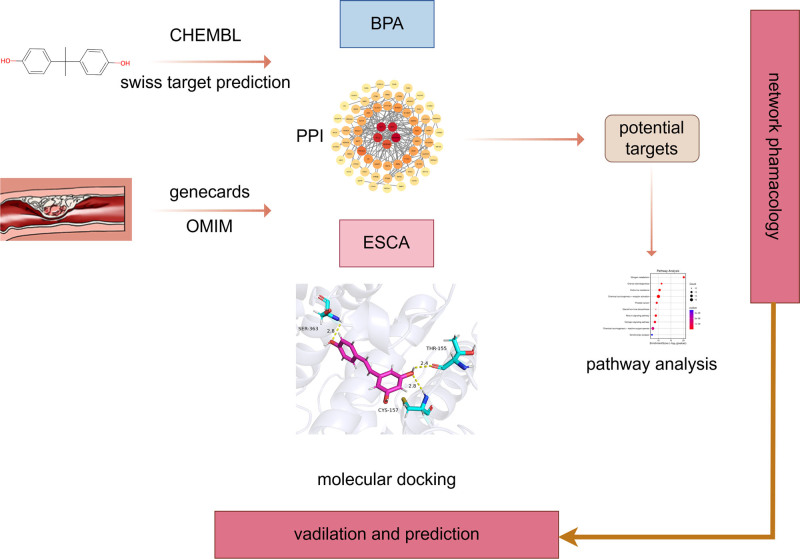
The study workflow of molecular mechanism of bisphenol A in promoting esophageal carcinoma. BPA = bisphenol A, ESCA = esophageal carcinoma, PPI = protein–protein interaction.

A growing body of evidence supports the carcinogenic potential of BPA^[15–17]^; however, most studies have focused on its association with reproductive system tumors, whereas research on other solid malignancies – particularly esophageal cancer – remains limited. Given that diet is the primary route of BPA exposure and that ingested BPA acts directly on the esophageal epithelium before undergoing hepatic–intestinal first-pass metabolism,^[18]^ the esophagus may be chronically affected by long-term, low-dose BPA exposure. In addition, large-scale transcriptomic resources such as The Cancer Genome Atlas (TCGA) and cBioPortal provide valuable datasets for validating the molecular mechanisms and genetic alterations of hub genes in ESCA. Integrating these multi-omics data with network toxicology and molecular docking analyses enables a more comprehensive evaluation of BPA’s potential carcinogenic role and prognostic relevance in ESCA. Therefore, this study employed a combined network toxicology–molecular docking–TCGA validation approach to systematically elucidate the potential molecular targets and mechanisms of BPA in ESCA, thereby providing a theoretical basis for understanding its carcinogenic effects and identifying potential intervention strategies.^[19,20]^ The overall research workflow is illustrated in Figure 1.

## 2. Materials and methods

### 2.1. Construction of the BPA target database

The standard SMILES string and molecular structure of BPA were retrieved from the PubChem database.^[21]^ Subsequently, potential human targets of BPA were identified using the ChEMBL database.^[22]^ To comprehensively identify targets, the Swiss Target Prediction tool (Swiss Institute of Bioinformatics [SIB], Lausanne, Switzerland) was also employed to detect any potentially missing targets.^[23]^ After cross-validating structural information to ensure consistency and accuracy, presumed targets corresponding to ChEMBL IDs were integrated, duplicates were removed, and gene names were standardized using the UniProt database.^[24]^ Finally, a comprehensive BPA target database was established.

### 2.2. Construction of the ESCA-related target network

The GeneCards and Online Mendelian Inheritance in Man databases were extensively searched using the keyword “esophageal carcinoma” to identify associated targets.^[25,26]^ To ensure high relevance between the identified genes, ESCA, and toxicity, a stringent median-based threshold was applied, retaining only genes above this value to construct a refined ESCA-related toxic target database. Venn diagram analysis was then performed to identify overlapping targets between the BPA and ESCA datasets, which were considered key candidate targets mediating the pro-carcinogenic effects of BPA in ESCA.

### 2.3. Construction of the protein–protein interaction (PPI) network and identification of key targets

Candidate targets mediating the BPA-induced effects in ESCA were imported into the STRING database to map their encoded proteins and interaction networks.^[27]^ During the analysis, the organism was set to “*Homo sapiens*,” with a minimum interaction score > 0.9 (highest confidence level) and a stringent false discovery rate (FDR) control applied. The STRING-derived data were imported into Cytoscape software (version 3.8.2; Cytoscape Consortium, San Diego) to compute topological parameters of nodes and edges, visualize molecular interactions, and construct the PPI network.^[28]^ Based on these results, topological analysis and scoring were performed using the MCC (Maximal Clique Centrality) algorithm in the CytoHubba plugin (National Taiwan University, Taipei, Taiwan) to identify central nodes and extract their subnetworks.^[29]^ Additionally, by integrating key topological parameters – degree centrality, betweenness centrality, and closeness centrality – the 5 targets with the highest cumulative values were selected as core candidates. To further screen and validate the expression profiles of these genes, differential expression analysis was conducted using GEO datasets comparing tumor and normal tissues, followed by the generation of expression distribution plots to identify significantly dysregulated genes in ESCA tissues. The gene expression dataset GSE20347 (from the GEO database) was analyzed to assess the differential expression of the 5 core targets identified through previous topological analysis (HSP90AA1, BCL2L2, AKT1, BRAF, and HSP90AB1), in order to further screen potential key genes. Differentially expressed genes between ESCA and normal tissues were defined as those with an absolute log_2_-fold change (|log_2_FC|) > 1 and an FDR-adjusted *P*-value < .05. Boxplots and violin plots were generated to compare expression levels of the 5 targets across tissue types, thereby identifying genes significantly dysregulated in ESCA.

### 2.4. Functional enrichment and pathway analysis

The DAVID database was used to perform gene ontology (GO) and Kyoto Encyclopedia of Genes and Genomes (KEGG) pathway enrichment analyses of these targets to explore the biological mechanisms underlying BPA-induced ESCA.^[30]^ The KEGG pathway analysis focused on pathways significantly associated with the pro-tumorigenic effects of BPA on ESCA. Results with a FDR < 0.05 were considered statistically significant, and the top 20 enriched terms from both GO and KEGG analyses were presented. This approach aimed to comprehensively elucidate the signaling pathways and biological processes (BP) involving key ESCA targets, clarify their underlying mechanisms, and visualize the enrichment results to facilitate clearer presentation and interpretation.

### 2.5. Molecular docking between BPA and key targets

To investigate the molecular interactions and binding modes between BPA and its key targets, molecular docking simulations were performed. For docking validation, the molecular structure of BPA was downloaded from the PubChem database in SDF format. Three-dimensional crystal structures of the target proteins were obtained from the Protein Data Bank (PDB) and saved in PDB format. Using PyMOL software (Schrödinger, LLC, New York), water molecules and ligands were removed from each protein, which was then saved in PDB format, and docking pocket parameters were determined using the GetBox plugin. The processed protein and ligand files were imported into AutoDockTools (version 1.5.6; The Scripps Research Institute, La Jolla) and converted into PDBQT format. Molecular docking was conducted using AutoDock Vina (version 1.1.2).^[31]^ Finally, the docking results were visualized using PyMOL (version 2.6.0) and Discovery Studio (version 2019; BIOVIA, Dassault Systèmes, San Diego) software.^[32]^

### 2.6. Validation using TCGA and cBioPortal databases

To further validate the expression and prognostic significance of the hub genes HSP90AA1 and HSP90AB1 in esophageal carcinoma (ESCA), RNA-seq and clinical data were obtained from The Cancer Genome Atlas (TCGA-ESCA) dataset via the GEPIA2 web platform (http://gepia2.cancer-pku.cn/).^[33]^ Box plots were generated to compare gene expression levels between primary tumor and normal tissues. Kaplan–Meier survival analyses were performed to assess the impact of HSP90AA1 and HSP90AB1 expression on overall survival (OS), and statistical significance was determined using the log-rank test (*P* < .05). Expression validation was further performed using the UALCAN portal (http://ualcan.path.uab.edu/).^[34]^ In addition, genetic alterations of HSP90AA1 and HSP90AB1 were analyzed using the cBioPortal database (https://www.cbioportal.org/),^[35]^ including mutation, amplification, and deletion frequencies. The correlation between HSP90AA1 and HSP90AB1 mRNA expression levels was further evaluated using Spearman correlation analysis in GEPIA2 based on TCGA-ESCA transcriptomic data. Correlation analysis was also verified using LinkedOmics (http://www.linkedomics.org/).

### 2.7. Ethical approval

This study used publicly available data and did not require ethical approval.

## 3. Results

### 3.1. Identification of BPA targets associated with ESCA

**Figure 2.**
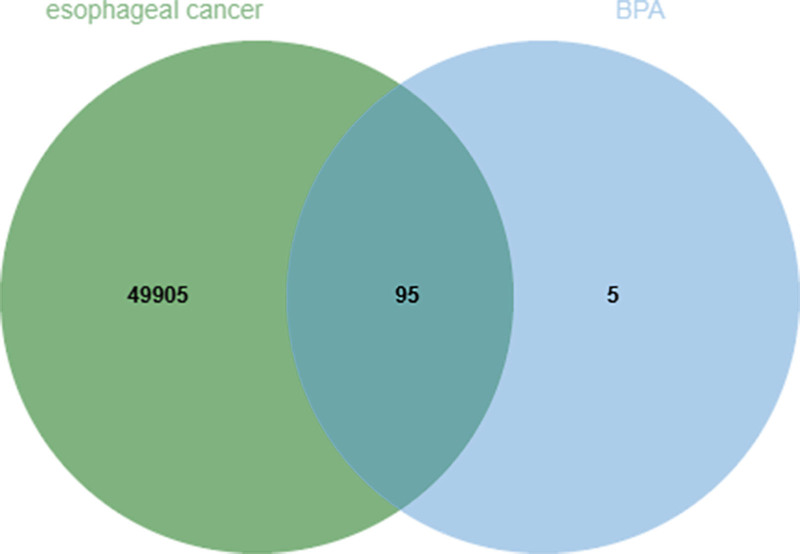
The intersection of potential BPA targets and ESCA-related genes. Venn analysis identified 95 overlapping targets, which were regarded as key candidate genes mediating the tumor-promoting effects of BPA on ESCA. BPA = bisphenol A, ESCA = esophageal carcinoma.

By integrating data from the ChEMBL and SwissTargetPrediction databases, 100 potential targets of BPA were identified. In addition, approximately 50,000 genes associated with ESCA were retrieved from the GeneCards and Online Mendelian Inheritance in Man databases. Venn diagram analysis revealed 95 overlapping targets, which were considered key candidate genes mediating the pro-tumorigenic effects of BPA on ESCA (Fig. 2).

### 3.2. PPI network analysis and identification of key targets

**Figure 3.**
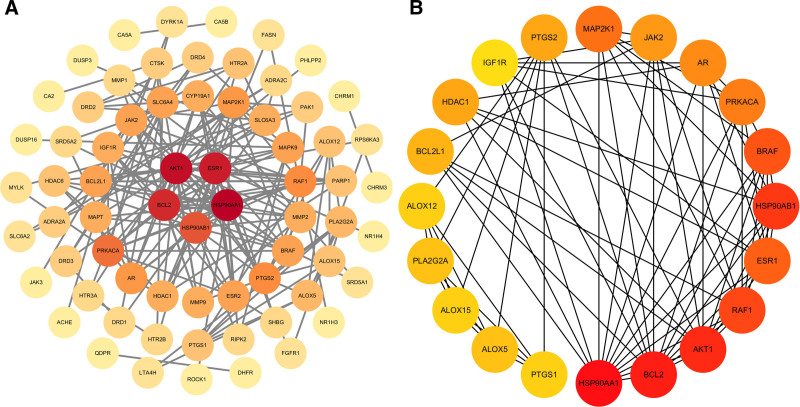
PPI network and candidate gene screening. (A) PPI network constructed using the STRING database (68 nodes, 178 edges), with a PPI enrichment test *P*-value < 1.0e−16. (B) The top 20 candidate key genes were identified using the MCC algorithm in CytoHubba, among which HSP90AA1, BCL2L2, AKT1, BRAF, and HSP90AB1 ranked in the top 5. PPI = protein–protein interaction.

After importing 95 the candidate genes into the STRING database, 68 genes were successfully mapped to form an interaction network, as several genes lacked known or predicted interaction information. The resulting network comprised 68 nodes and 178 edges, with a PPI enrichment *P*-value < 1.0e−16, indicating significant biological relevance (Fig. 3A).

**Figure 4.**
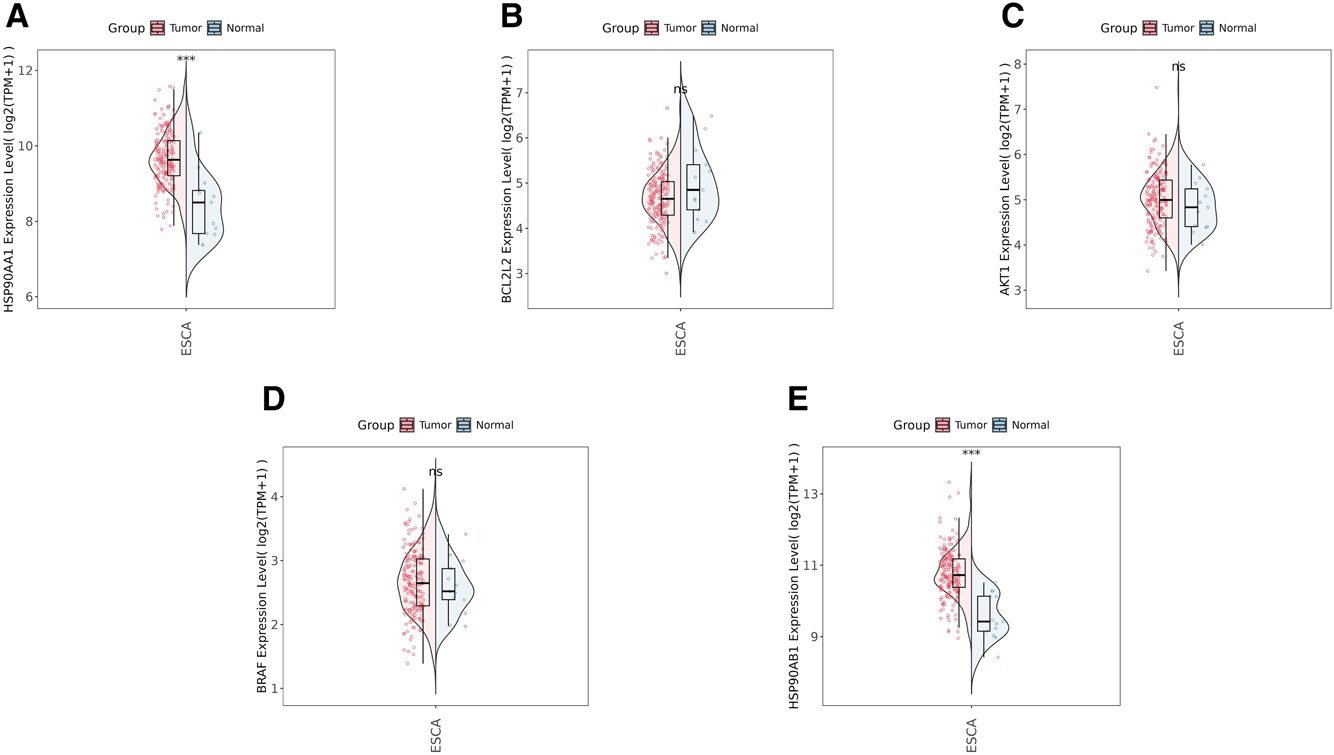
Validation of candidate gene expression in the GSE20347 dataset. (A) HSP90AA1, (B) BCL2L2, (C) AKT1, (D) BRAF, and (E) HSP90AB1 expression levels in ESCA versus normal tissues. HSP90AA1 and HSP90AB1 were significantly overexpressed, whereas BCL2L2, AKT1, and BRAF showed no significant differences. ESCA = esophageal carcinoma.

Further analysis using the MCC algorithm in the CytoHubba plugin ranked the network nodes and identified the top 20 key genes based on centrality scores (Fig. 3B). According to network density and centrality metrics, the 5 core targets with the highest connectivity were identified as HSP90AA1, BCL2L2, AKT1, BRAF, and HSP90AB1. Subsequently, the gene expression dataset GSE20347 was used to validate the differential expression of these 5 candidate genes. The analysis revealed that only HSP90AA1 and HSP90AB1 were significantly overexpressed in ESCA tissues compared with normal tissues, whereas BCL2L2, AKT1, and BRAF showed no significant differences in expression (Fig. 4).

### 3.3. Functional annotation and pathway enrichment analysis

**Figure 5.**
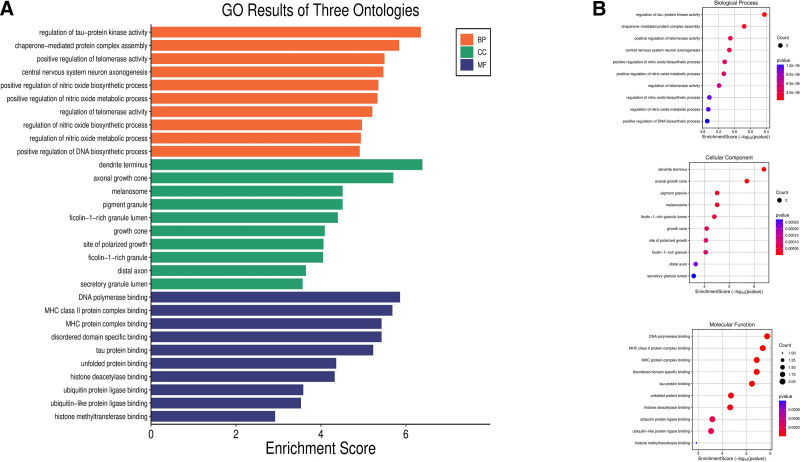
GO enrichment analysis results. (A) The bar plot displays the GO enrichment results for the 3 ontologies (BP, CC, and MF), where colors represent ontology categories and bar length indicates the enrichment score. (B) The bubble plot further visualizes enriched GO terms for each ontology, with the *x*-axis showing the enrichment score (−log10 *P*-value), the *y*-axis representing specific GO terms, bubble size indicating the number of genes, and color reflecting the level of significance. BP = biological process, CC = cellular component, GO = gene ontology, MF = molecular function.

GO analysis of 95 potential targets revealed numerous significantly enriched GO terms across BP (217 terms), cellular components (CC, 34 terms), and molecular functions (MF, 26 terms). After ranking GO terms by FDR values, the 10 most significantly enriched terms in each category were visualized as enrichment charts (Fig. 5A). The analysis, illustrated in Figure 5B, provides a detailed overview of the functional characteristics and subcellular localization patterns of BPA-related genes and proteins. Specifically, the BP terms were primarily associated with nervous system development and signal regulation, telomerase activity, and nitric oxide metabolism, suggesting that these genes may play crucial roles in neuronal development, cellular stress responses, and the maintenance of telomere and nitric oxide homeostasis, thereby influencing neural and disease-related processes. The CC terms were primarily enriched in cellular structures such as dendritic terminals, axon growth cones, melanosomes, and secretory granules, indicating their close association with neuronal structure formation, signal transmission, and intracellular transport. The MF terms were significantly enriched in DNA polymerase binding, MHC complex binding, tau protein binding, histone modification, and ubiquitination, highlighting critical roles in DNA repair and replication, immune regulation, epigenetic modulation, and protein degradation. Overall, these findings underscore the multifunctional roles of the candidate genes in neural development, signal regulation, and immune–epigenetic crosstalk.

**Figure 6.**
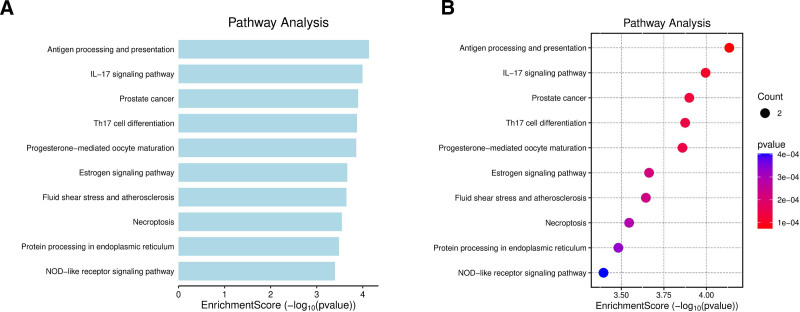
Pathway enrichment analysis. (A) The bar plot displays significantly enriched KEGG pathways, with the *x*-axis representing the enrichment score and the *y*-axis showing specific pathway names. (B) The bubble plot further visualizes the enriched pathways, where the *x*-axis indicates the enrichment score (−log10 *P*-value), the *y*-axis lists pathway names, bubble size represents the number of genes, and color denotes the level of significance. KEGG = Kyoto Encyclopedia of Genes and Genomes.

Furthermore, KEGG pathway analysis of the 95 potential targets using the DAVID database identified 14 significantly enriched pathways. These targets were significantly enriched in several critical pathways, including antigen processing and presentation, the IL-17 signaling pathway, prostate cancer, and Th17 cell differentiation, which are implicated in immune regulation, inflammatory responses, and tumorigenesis. In addition, enrichment was observed in progesterone-mediated oocyte maturation, estrogen signaling, and NOD-like receptor signaling pathways, all closely related to endocrine and inflammatory regulation. Notably, significant enrichment of pathways such as fluid shear stress and atherosclerosis, necroptosis, and protein processing in the endoplasmic reticulum suggests that these genes may participate in cellular stress responses and programmed cell death. Collectively, these findings highlight the crucial roles of the candidate genes in immune regulation, tumor progression, and cellular homeostasis, providing valuable insights for identifying potential therapeutic targets and biomarkers (Fig. 6).

### 3.4. Molecular docking analysis of BPA with key targets

**Figure 7.**
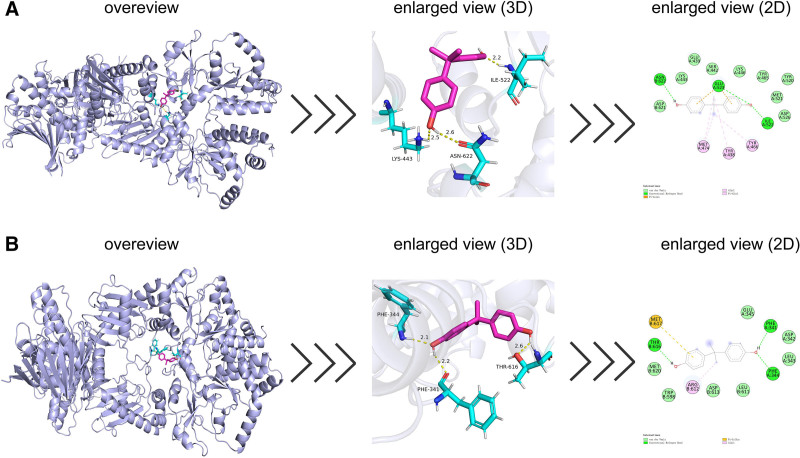
Molecular docking results showing the lowest binding energy conformations between BPA and target proteins. (A) BPA–HSP90AA1 complex. (B) BPA–HSP90AB1 complex. BPA = bisphenol A.

To explore the interaction mechanisms between BPA and its key targets, comprehensive molecular docking simulations were performed. Docking simulations were performed for the 2 core targets, HSP90AA1 and HSP90AB1, using AutoDock software. All targets exhibited binding energies below −5.0 kcal/mol, indicating strong binding affinities between BPA and the 2 core targets. These results suggest that BPA can spontaneously and stably bind to these core targets, thereby contributing to the molecular mechanisms underlying its tumor-promoting effects in ESCA. Using Discovery Studio and PyMOL, the lowest-energy binding conformations between BPA and each core target were visualized to illustrate their interaction patterns (Fig. 7), and the corresponding binding energy results are summarized in Table 1.

**Table 1:** Molecular docking binding energies between BPA and key target proteins (HSP90AA1 and HSP90AB1).

Small molecule	Target protein	Binding energy, kcal/mol
BPA	HSP90AA1	−7.5
	HSP90AB1	−7.0

The table summarizes the molecular docking binding energies between BPA and the 2 key target proteins (HSP90AA1 and HSP90AB1). Binding energy is expressed in kilocalories per mole (kcal/mol); negative values indicate attractive interactions between BPA and the target protein, with larger absolute values representing stronger binding affinities.

BPA = bisphenol A.

### 3.5. TCGA validation of hub genes

**Figure 8.**
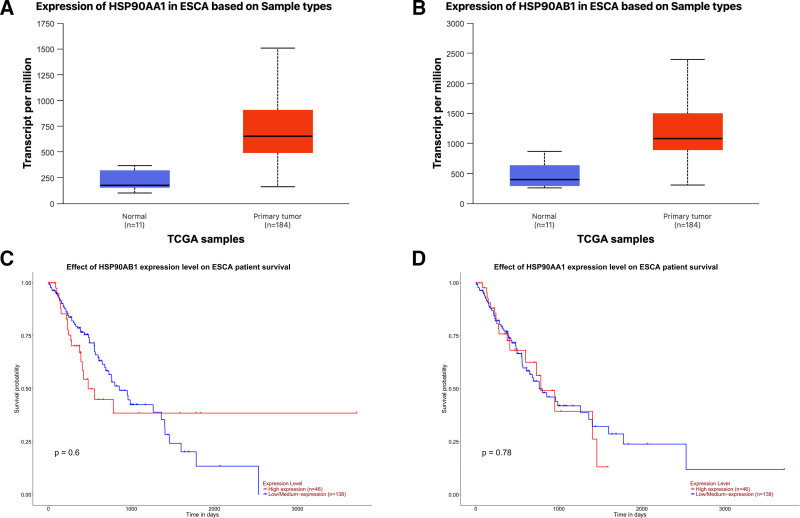
Expression and survival analyses of HSP90AA1 and HSP90AB1 in ESCA based on TCGA. (A) Expression levels of HSP90AA1 in ESCA and normal tissues. (B) Expression levels of HSP90AB1 in ESCA and normal tissues. (C) Kaplan–Meier survival curve showing the association between HSP90AB1 expression and overall survival (*P* = .60). (D) Kaplan–Meier survival curve showing the association between HSP90AA1 expression and overall survival (*P* = .78). Both genes were markedly upregulated in ESCA tissues, but their expression levels were not significantly correlated with overall survival in the TCGA–ESCA cohort. ESCA = esophageal carcinoma, TCGA = The Cancer Genome Atlas.

**Figure 9.**
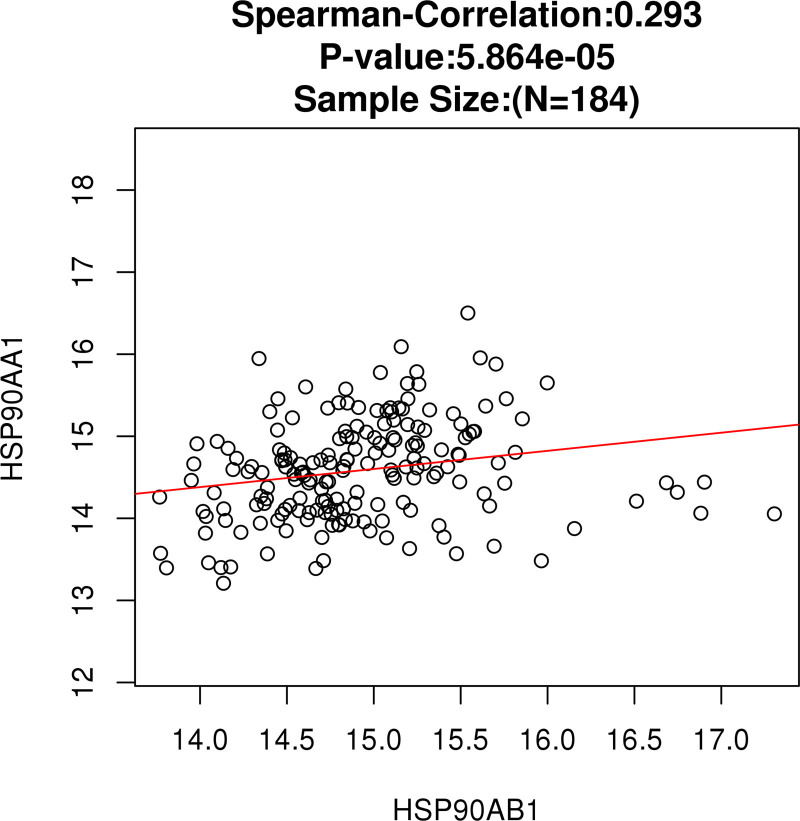
Correlation analysis between HSP90AA1 and HSP90AB1 expression in ESCA based on TCGA. Scatter plot showing the positive correlation between HSP90AA1 and HSP90AB1 expression levels in the TCGA–ESCA dataset (LinkedOmics, Spearman *R* = 0.293, *P* = 5.86 × 10^−5^). The significant positive correlation suggests potential co-regulation between the 2 HSP90 isoforms in the development of esophageal carcinoma. ESCA = esophageal carcinoma, TCGA = The Cancer Genome Atlas.

Kaplan–Meier survival analyses based on the TCGA-ESCA dataset (Fig. 8C and D) showed no statistically significant differences in overall survival between high- and low-expression groups of HSP90AA1 (*P* = .78) and HSP90AB1 (*P* = .60). Nevertheless, both genes were significantly upregulated in ESCA tissues compared with normal tissues (Fig. 8A and B), indicating that they may play tumor-promoting roles at the transcriptional level, although their prognostic impact requires further validation in larger cohorts. Correlation analysis revealed a significant positive association between HSP90AA1 and HSP90AB1 expression levels (Spearman *R* = 0.293, *P* = 5.86 × 10^−5^; Fig. 9), suggesting potential co-expression and functional interaction between these 2 HSP90 isoforms.

**Figure 10.**
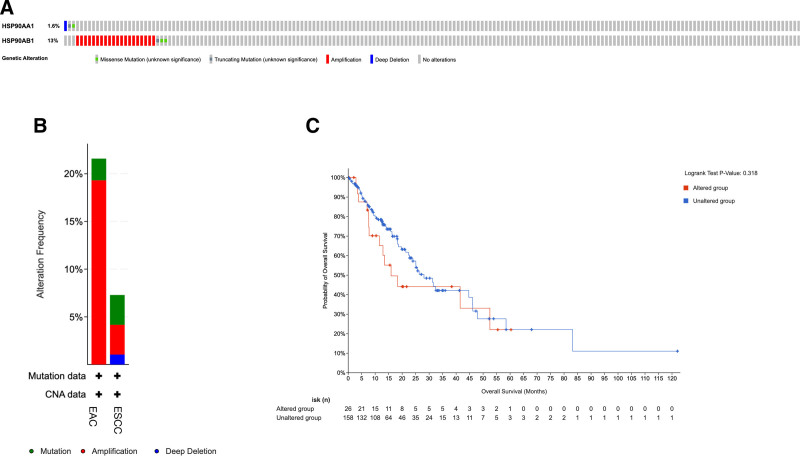
Genetic alterations and prognostic impact of HSP90AA1 and HSP90AB1 in ESCA based on cBioPortal. (A) Distribution of gene alterations (mutation, amplification, and deletion) in HSP90AA1 and HSP90AB1 within the TCGA–ESCA cohort. (B) Alteration frequencies of HSP90AA1 and HSP90AB1 in esophageal squamous carcinoma (ESCC) and adenocarcinoma (EAC) subtypes. (C) Kaplan–Meier curve comparing overall survival between patients with and without HSP90AA1 or HSP90AB1 alterations in TCGA–ESCA. HSP90AB1 showed a higher alteration frequency (13%) than HSP90AA1 (1.6%), mainly due to amplification events. Although not statistically significant (*P* = .318), patients harboring HSP90AB1 alterations exhibited a trend toward poorer overall survival. Data from TCGA–ESCA (n = 162 tumor, n = 11 normal tissues). ESCA = esophageal carcinoma, TCGA = The Cancer Genome Atlas.

Furthermore, cBioPortal analysis indicated that HSP90AB1 exhibited a higher alteration frequency (13%) than HSP90AA1 (1.6%), predominantly through gene amplification (Fig. 10A and B). Although the overall survival difference between altered and unaltered groups did not reach statistical significance (*P* = .318; Fig. 10C), patients with HSP90AB1 amplification showed a trend toward poorer prognosis.

Collectively, these findings support the oncogenic roles of HSP90AA1 and HSP90AB1 and highlight their potential as diagnostic and prognostic biomarkers in ESCA.

## 4. Discussion

ESCA is a highly aggressive malignancy of the digestive system, characterized by genetic heterogeneity, epigenetic abnormalities, and dysregulation of multiple critical signaling pathways.^[36]^ Although therapeutic strategies have advanced in recent years, the overall prognosis of patients remains poor, indicating an urgent need to explore novel pathogenic factors and potential therapeutic targets from the perspectives of environmental exposure and molecular mechanisms. BPA is a widely distributed environmental endocrine disruptor with estrogen-like activity and multiple biological targets. Both epidemiological and experimental studies suggest that BPA is involved in the initiation and progression of multiple cancers.^[37]^ However, the mechanistic role of BPA in ESCA remains largely unexplored. Therefore, an integrative approach combining network toxicology and molecular docking was employed to elucidate the potential molecular mechanisms of BPA in ESCA, thereby providing new evidence for understanding its tumor-promoting effects.

The TCGA-based validation further confirmed the clinical significance of these findings. Both HSP90AA1 and HSP90AB1 were markedly upregulated in ESCA tissues; however, survival analyses based on TCGA data did not reveal statistically significant differences in overall survival between high- and low-expression groups. This may be due to the limited sample size and the heterogeneity of ESCA subtypes (ESCC vs EAC) within TCGA. Moreover, mRNA expression does not necessarily reflect protein activity, which often depends on posttranslational modifications and client-protein interactions of HSP90. Therefore, the overexpression pattern combined with strong binding affinities to BPA still supports the hypothesis that BPA may promote ESCA through HSP90-mediated molecular chaperone mechanisms, while larger-scale and protein-level studies are warranted. Although statistical significance was not observed in survival analysis, the consistent overexpression patterns and strong molecular docking interactions support the potential involvement of HSP90AA1 and HSP90AB1 in BPA-mediated carcinogenic mechanisms.

By integrating multi-database data, 95 potential targets of BPA in ESCA were identified, laying the foundation for subsequent PPI network construction and enrichment analyses. The PPI network and differential expression analyses further identified HSP90AA1 and HSP90AB1 as key molecular targets. Previous studies have also demonstrated that BPA can modulate the expression or activity of the 2 heat shock proteins HSP90AA1^[38,39]^ and HSP90AB1.^[40]^ HSP90AB1 is overexpressed in multiple cancers and promotes tumor cell proliferation, invasion, and drug resistance by stabilizing oncoproteins, enhancing angiogenesis, and regulating telomerase activity and key signaling pathways, thereby representing a potential therapeutic target.^[41]^ In ESCA, HSP90AB1 has been incorporated into autophagy-related prognostic gene models,^[14]^ suggesting that it may serve as a potential prognostic biomarker. Mechanistic studies have revealed that the deubiquitinase JOSD2 stabilizes HSP90AB1 through deubiquitination, thereby activating oncogenic signaling pathways such as MAPK/ERK and PI3K/AKT, ultimately promoting tumor proliferation, migration, and drug resistance.^[12]^ Meanwhile, HSP90AA1 also plays a crucial role in ESCA. Genetic studies have shown that expression quantitative trait loci (eQTLs) of HSP90AA1 are significantly associated with the risk of Barrett esophagus and esophageal adenocarcinoma, suggesting that it may represent a genetic susceptibility gene contributing to disease development.^[42]^ Transcriptomic and machine learning analyses have identified HSP90AA1 as a pivotal hub gene, whose high expression is strongly correlated with immune infiltration and cell death, significantly enhancing the diagnostic accuracy of predictive models (AUC = 0.997).^[43]^ Moreover, the abnormal activation of HSP90AA1 is driven by super-enhancers, and inhibition of this protein effectively suppresses tumor cell proliferation and migration, underscoring its potential as a therapeutic target.^[11]^ Combined with the molecular docking results, these findings suggest that BPA may promote ESCA progression by stably binding to HSP90AA1 and HSP90AB1, thereby interfering with chaperone-dependent molecular mechanisms.

GO and KEGG enrichment analyses further revealed that BPA-related targets were significantly enriched in BP terms such as nervous system development, telomerase activity, and nitric oxide metabolism, which are closely associated with cellular stress responses, chromosomal stability, and tumor cell adaptability. At the CC level, the targets were primarily localized to dendritic terminals, axon growth cones, melanosomes, and secretory granules, suggesting roles in cell signaling, intracellular transport, and tumor microenvironment formation. At the MF level, the targets were significantly enriched in DNA polymerase binding, MHC complex binding, histone modification, and ubiquitination, which are closely related to DNA repair and replication, immune responses, and epigenetic regulation. Previous studies have shown that BPA alters chromatin states by upregulating epigenetic regulators such as EZH2, thereby increasing tumor susceptibility,^[6]^ which is consistent with these findings. Notably, KEGG pathway analysis identified enrichment in immune-related pathways, including IL-17 signaling, Th17 cell differentiation, and antigen processing and presentation. Animal studies have shown that BPA exposure increases IL-17 levels, elevates the Th17-cell proportion, and decreases Treg-cell levels, disrupting immune homeostasis via mechanisms linked to PI3K/AKT/mTOR activation.^[44]^ Further evidence indicates that this Th17/Treg imbalance is associated with autophagy dysfunction, characterized by upregulation of mTOR and p62 and downregulation of LC3.^[45]^ Moreover, BPA can trigger pyroptosis and other pro-inflammatory cell death pathways by inducing ROS accumulation and activating the NLRP3 inflammasome,^[46]^ thereby disrupting immune homeostasis within the tumor microenvironment. Collectively, these findings suggest that the tumor-promoting effects of BPA in ESCA extend beyond estrogenic activity and involve complex multilevel interactions among immune, inflammatory, epigenetic, and autophagic mechanisms.

From a clinical perspective, as a ubiquitous chemical in everyday environments, BPA poses a potential health threat through long-term, low-dose exposure. In light of these findings, enhanced risk assessment and public-health interventions targeting BPA exposure are particularly warranted in regions with a high incidence of esophageal cancer. Meanwhile, the central roles of HSP90AA1 and HSP90AB1 suggest that they may serve as novel molecular targets for therapeutic intervention. Previous studies have shown that HSP90 inhibitors such as 17-AAG, Ganetespib, and AUY922 significantly suppress AKT/ERK signaling, reduce MYC expression, and enhance radiosensitivity in ESCC models.^[47–49]^ Therefore, a combined strategy integrating BPA exposure control with HSP90-targeted therapy may provide a novel avenue for the prevention and treatment of ESCA. By integrating network toxicology, molecular docking, and multi-omics validation from TCGA and cBioPortal, this study bridges mechanistic and clinical evidence for BPA-induced esophageal carcinogenesis.

Nevertheless, this study has several limitations. First, the results primarily rely on public databases and computational simulations and lack experimental validation. Second, the specific carcinogenic effects of BPA metabolites remain to be elucidated. Finally, large-scale epidemiological evidence on the dose–response relationship between long-term, low-dose exposure and esophageal cancer risk remains insufficient. Therefore, future studies should integrate multi-omics analyses, cellular and animal models, and epidemiological investigations to comprehensively elucidate the tumor-promoting role of BPA in ESCA and evaluate its feasibility as a clinical intervention target.

## 5. Conclusions

In conclusion, through an integrated analysis combining network toxicology, molecular docking, and TCGA validation, this study revealed the potential molecular mechanisms of BPA in ESCA. The results demonstrated that BPA may act on core targets such as HSP90AA1 and HSP90AB1 to modulate key oncogenic and immune–inflammatory pathways, potentially contributing to ESCA progression. The findings suggest that BPA is not only a common environmental endocrine disruptor but also a potential risk factor driving the initiation and progression of ESCA.

The findings provide new insights into the carcinogenic mechanisms of BPA and offer a potential theoretical basis for the prevention and treatment of ESCA. Future research should validate the effects of BPA through cellular and animal experiments, studies on its metabolites, and large-scale epidemiological investigations. Moreover, molecular interventions targeting key proteins such as HSP90 – particularly combining HSP90 inhibitors with existing therapeutic approaches – may offer novel strategies to improve the prognosis of patients with ESCA.

## Acknowledgments

We thank the supporting institutions and acknowledge TCGA, GEPIA2, UALCAN, cBioPortal, and GEO for data resources.

## Author contributions

**Conceptualization:** Ming Hou, Cheng Wang.

**Data curation:** Ming Hou, Chongyang Liu.

**Formal analysis:** Ming Hou.

**Investigation:** Hanbing Jia, Chongyang Liu.

**Methodology:** Hanbing Jia.

**Project administration:** Cheng Wang.

**Resources:** Cheng Wang.

**Software:** Chongyang Liu.

**Supervision:** Cheng Wang.

**Visualization:** Ming Hou.

**Writing – original draft:** Ming Hou.

**Writing – review & editing:** Ming Hou, Hanbing Jia.
